# High doses of lercanidipine are better tolerated than other dihydropyridines in hypertensive patients with metabolic syndrome: results from the TOLERANCE study

**DOI:** 10.1111/j.1742-1241.2008.01736.x

**Published:** 2008-05-01

**Authors:** V Barrios, C Escobar, M de la Figuera, J Honorato, J L Llisterri, J Segura, A Calderón

**Affiliations:** 1 Hospital Ramón y Cajal Madrid, Spain; 2 Hospital Infanta Sofía Madrid, Spain; 3 CAP La Mina, San Adrían del Besós Barcelona, Spain; 4 Clínica Universitaria de Navarra Navarra, Spain; 5 CS Joaquín Benlloch Valencia, Spain; 6 Hospital 12 de Octubre Madrid, Spain; 7 CS Rosa de Luxemburgo, SS de los Reyes Madrid, Spain

## Abstract

**Aims/Introduction:**

The TOlerabilidad de LERcanidipino 20 mg frente a Amlodipino y **N**ifedipino en **C**ondicion**E**s normales de uso study was aimed to compare the tolerability of high doses of lercanidipine with amlodipine and nifedipine gastro-intestinal therapeutic system (GITS) in the treatment of hypertension in daily clinical practice.

**Patients/methods:**

Essential hypertensives ≥ 18 years, treated during at least 1 month with lercanidipine 20 mg, amlodipine 10 mg or nifedipine GITS 60 mg, after a previous treatment course of at least 1 month with half the dose of the corresponding drugs were included. We present the data of the subgroup of patients with metabolic syndrome (MetS).

**Results:**

Three hundred and thirty-seven of the 650 study population fulfilled criteria of MetS, 233 (69.1%) on lercanidipine and 104 (30.9%) on amlodipine/nifedipine GITS. Overall, a significantly lower proportion of lercanidipine-treated patients showed adverse reactions (ARs) when compared with patients receiving other-dihydropyridines (DHPs) (60.1% vs. 73.1%, p = 0.003). Similarly, the most common vasodilation-related ARs (oedema, swelling, flushing and headache) were significantly less frequent in lercanidipine group (all p < 0.01).

**Conclusion:**

In conclusion, lercanidipine appears to exhibit a better tolerability profile and less vasodilation-related ARs compared with other DHPs in hypertensive patients with MetS.

**What's known**

Only a small proportion of hypertensive patients with MetS attain BP goals. The poor treatment compliance may in part explain this poor BP control. One of the most important reasons for this inadequate compliance is the presence of adverse events related to antihypertensive therapy. CCB are drugs widely used for the treatment of hypertension. However, tolerability of different CCB may differ, especially in those patients at high risk, such as those with MetS.

**What's new**

In a large sample of hypertensive patients with metabolic syndrome recruited and managed in conditions of daily practice, this study shows that treatment with lercanidipine at high doses is associated with a lower rate of adverse events related to vasodilation compared with high doses of amlodipine or nifedipine GITS.

## Introduction

The metabolic syndrome (MetS) is characterised by an array of cardiovascular risk factors which may be predictive of longer-term disease consequences in certain individuals. The syndrome is associated with insulin resistance and hyperglycaemia, and is associated with an increased risk of developing type 2 diabetes and cardiovascular disease (1,2). The prevalence of MetS is rising in Western countries because of a progressive increase in the proportion of patients with diabetes and obesity (3).

The hypertensive population with MetS represents a particularly high-risk group because of the increased incidence of cardiovascular complications (4–8). In fact, current European Guidelines have included the MetS as an important component of the risk stratification in patients with hypertension, as it markedly increases cardiovascular risk (8). Blood pressure (BP) control is essential in this population to improve prognosis. But, only a small proportion of hypertensive patients with MetS attain BP goals (9). The poor treatment compliance may in part explain this poor BP control. One of the most important reasons for this inadequate compliance is the presence of adverse events related to antihypertensive therapy. This is particularly important in these patients, moreover, taking into account that they usually need several drugs to achieve BP objectives, that increases the possibility of causing side effects (9,10).

Calcium channel blockers (CCB) are drugs widely used for the treatment of hypertension. Lercanidipine is a highly lipohilic third generation dihydropyridine (DHP) (11). Its efficacy has been evaluated in non-comparative (12–16) and comparative studies (17–19). Lercanidipine is generally well tolerated during monotherapy in patients with mild-to-moderate hypertension even when compared with other DHPs (12,14,20). Nonetheless, this information is generally provided by clinical trials with commonly strict selection criteria with less information available from daily clinical practice. Moreover, as in most trials the starting dose was 10 mg qd, scarce data were available with higher doses. Although lercanidipine has been compared with other antihypertensive drugs in high-risk populations such as elderly people, diabetics and patients with renal impairment, to date there is no information about the tolerability and efficacy of this drug in hypertensive subjects with MetS in daily clinical practice (19–21).

The TOlerabilidad de LERcanidipino 20 mg frente a Amlodipino y **N**ifedipino en **C**ondicion**E**s normales de uso (TOLERANCE) study was aimed to compare the tolerability, with special emphasis on vasodilation-related adverse reactions (ARs), of high doses of lercanidipine with other DHP (amlodipine and nifedipine GITS) also given at daily high doses in conditions of common clinical practice (22). In this paper, we present the data related to the subgroup of patients with MetS from the TOLERANCE study database.

## Patients and methods

The TOLERANCE was an observational, cross-sectional and multicentre study performed in Primary Care Centres from all around Spain. Outpatients aged ≥ 18 years, of both genders, with essential hypertension who had been treated at least for 1 month with lercanidipine, amlodipine or nifedipine GITS at low doses (10, 5 and 30 mg daily respectively) and who were titrated to higher doses of the same drugs (20, 10 and 60 mg respectively) because of an uncontrolled BP in a 2 : 1 design were included (22). The choice of the CCB was based on the physicians’ decision, according to their own clinical criteria.

Blood pressure readings were taken with a mercury sphygmomanometer or validated automatic devices where available with the patient in a seated position and the back supported, and after resting 5 min. Two measurements were taken by physicians following the current guidelines and the mean was recorded (8). Adequate BP control was defined as systolic BP < 140 mmHg and diastolic BP <90 mmHg (< 130 and < 80 mmHg for diabetics) (23). As this study was aimed to reflect clinical practice, when BP control was not attained after lercanidipine, amlodipine or nifedipine GITS at high doses, the investigators could freely add more antihypertensive medication. Patients underwent a complete physical examination, and they should have a complete blood test (haematology and biochemistry with a lipid profile) performed within the last 3 months. Waist circumference was measured at the midway point between the iliac crest and the costal margin. MetS was diagnosed according to NCEP-ATP III criteria, requiring the presence of three or more of the following: abdominal obesity (waist circumference > 102/88 cm or > 40/35 inches for men/women); triglycerides ≥ 150 mg/dl; high-density lipoprotein cholesterol < 40/50 mg/dl (men/women); fasting glucose ≥ 110 mg/dl or BP ≥ 130/85 mmHg (24).

Adverse reactions were spontaneously reported by the patient or elicited using a 16-item checklist similar to the one used in the COHORT trial (20) including those symptoms considered related to vasodilation and the most commonly adverse events reported during registration trials. The study was conducted according to good clinical practice guidelines and was approved by the local Clinical Research Ethic Committee. All participants provided a written informed consent to take part in the study.

### Statistical analysis

The primary variable of the study was evaluated through the frequency of ankle oedema and other vasodilation-related adverse events according to the questionnaire used in the study. Secondary end-points were frequency of spontaneously adverse events notified by the patient, rates of BP control and percentage of patients classified as good compliers according to the Haynes–Sacket test (25). Continuous variables were averaged and expressed as means ± standard deviation. Categorical items were expressed as per cent frequency; 95% confidence intervals were provided when necessary. Differences between means of different parameters were compared by the Student *t*-test. Differences between percentages were compared with the chi-squared test. Categorical data were also analysed with this test. p < 0.05 was used as the level of statistical significance. A logistic regression analysis was performed to determine what factors could influence the incidence of adverse events related to vasodilation (dependent variable). Clinical characteristics of study population, cardiovascular risk factors, target organ damage, associated clinical conditions, antihypertensive treatments, concomitant treatments and biochemical parameters were included as independent variables in the logistic regression analysis.

## Results

A total of 61 investigators recruited 650 patients (67.4 ± 11.1 years; 47% male) in the overall TOLERANCE study. A total of 337 patients (52%) of the study population fulfilled criteria of MetS, of whom 233 (69.1%) were taking lercanidipine and 104 (30.9%) amlodipine/nifedipine GITS. Clinical characteristics of the patients were similar between both groups, although there was a trend to more diabetes and more obesity (p = 0.13 and p = 0.26 respectively) in lercanidipine group (Table 1).

**Table 1:** Clinical characteristics of study population

	Lercanidipine	Amlodipine/ nifedipine	p
Age (years)	65.1 ± 11	66.1 ± 10	ns
Gender (female)	57.3%	58.7%	ns
Diabetes	43.2%	33.7%	ns
Hypercholesterolaemia	58.5%	60.4%	ns
Sedentary life style	72.7%	75.0%	ns
Obesity (BMI ≥ 30 kg/m^2^)	63.3%	56.6%	ns
History of heart disease	61.8%	62.4%	ns
Time of evolution of hypertension (months)	68.8 ± 14	70.7 ± 15	ns

Data were expressed as mean ± standard deviation or percentages. ns, not significant (p > 0.05); BMI, body mass index.

The changes in BP and heart rate along the study are shown in Table 2. There was a significant decrease of BP values between high- and low-dose treatment in each group, but without differences between both groups. The percentage of patients with an adequate BP control was 41.4% in the lercanidipine group and 35.0% in the amlodipine/nifedipine group (p = ns). The proportion and type of concomitant antihypertensive drugs were similar in both groups (46.4% in lercanidipine group needed additional antihypertensive to achieve BP goal vs. 53.9% in amlodipine/nifedipine GITS group, p = ns) (Table 3). There were no significant differences between both groups in biochemical parameters.

**Table 2:** Blood pressure and heart rate along the study

	Low dose			High dose			
			
	Lercanidipine	Amlodipine/nifedipine	p*	Lercanidipine	Amlodipine/nifedipine	p†	p‡
SBP	157.9 ± 14.0	157.8 ± 10.4	ns	144.4 ± 12.9	145.0 ± 11.4	ns	< 0.05
DBP	92.7 ± 6.7	92.4 ± 7.3	ns	83.3 ± 6.4	84.5 ± 7.1	ns	< 0.05
HR	78.7 ± 8.2	78.5 ± 7.1	ns	77.1 ± 7.6	77.2 ± 7.8	ns	ns

* p between lercanidipine and amlodipine/nifedipine groups at low dose.

† p between lercanidipine and amlodipine/nifedipine groups at high dose.

‡ p between high vs. low dose. SBP, systolic blood pressure (mmHg); DBP, diastolic blood pressure (mmHg); HR, heart rate (bpm); ns, not significant.

**Table 3:** Concomitant antihypertensive therapy

Drugs	Lercanidipine (%)	Amlodipine/ nifedipine (%)	p
Diuretics	24.0	23.1	ns
ARB	16.3	20.2	ns
ACE inhibitors	11.6	17.3	ns
Beta blockers	5.2	7.7	ns
Alpha blockers	3.4	3.9	ns
Others	0.9	1.0	ns

ARB, angiotensin receptor blockers; ACE, angiotensin-converting enzyme; ns, not significant.

**Figure 1:**
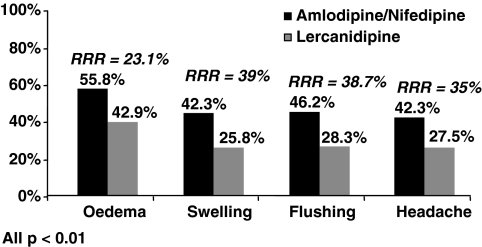
Risk reduction for the most frequent vasodilatation-related adverse events

With regard to the side effects reported by the questionnaire at high doses, patients treated with lercanidipine showed a lower proportion when compared with amlodipine/nifedipine GITS group (60.1% vs. 73.1%, respectively, p = 0.003). Similarly, lercanidipine vs amlodipine/nifedipine GITS showed a better tolerability profile at low doses (39.9% vs. 54.8%, respectively, p = 0.02). Table 4 shows the distribution between groups of drug-related signs and symptoms according to the checklist. The most frequent adverse event was ankle oedema in both groups. Almost all side effects were more prevalent in amlodipine/nifedipine GITS group. The Figure 1 shows the risk reduction for the most frequent vasodilatation-related adverse events. The classification of the severity of ARs is shown in Table 5. According to the Haynes–Sackett test, the percentage of patients considered as good compliers was similar in both groups (93.7% lercanidipine vs. 92.7% in amlodipine/nifedipine, p = ns). Concerning the changes in antihypertensive treatment made by the investigators, in 91.5% of patients in the lercanidipine group the treatment was maintained, whereas in the amlodipine/nifedipine group only 55.9% did not changed their treatment regimen (p < 0.001).

**Table 4:** Distribution of signs and symptoms according to the checklist with dihydropyridines given at high doses

Signs/symptoms	Global (%)	Lercanidipine (%)	Amlodipine/ nifedipine (%)	p
Ankle oedema	46.9	42.9	55.8	0.007
Flushes	33.8	28.3	46.2	< 0.001
Headache	32.0	27.5	42.3	0.002
Swelling	30.9	25.8	42.3	< 0.001
Fatigue	19.3	15.9	26.9	0.01
Dizziness	16.0	14.2	20.2	ns
Palpitations	14.2	10.7	22.1	0.006
Constipation/diarrhoea	13.9	10.3	22.1	0.005
Pyrosis	11.3	8.6	17.3	0.02
Sexual dysfunction	10.4	8.2	15.4	0.04
Dyspnoea	9.8	7.7	14.4	ns
Blurred vision	9.2	8.2	11.5	ns
Skin rush	8.0	4.7	15.4	0.001
Thoracic pain	5.3	6.0	3.8	ns
Gum swelling	2.4	1.7	3.8	ns
Thoracic swelling	1.8	1.3	2.9	ns

ns, not significant (p > 0.05).

**Table 5:** Distribution of severity of adverse events according to antihypertensive therapy

Severity of adverse events	Lercanidipine	Amlodipine/ nifedipine	p
Mild (%)	72.9	56.6	0.03
Moderate (%)	25.0	40.8	0.04
Severe (%)	2.1	2.6	ns

A logistic regression analysis was performed to examine which factors could influence the appearance of adverse events related to vasodilatation. The use of diuretics and the history of cardiac disease were related to the presence of side effects [odds ratio 2.93, 95% CI: 1.29–6.65 and 8.20 (2.45–27.43) respectively], and treatment with lercanidipine of a lower proportion of ARs [0.44 (0.23–0.85)].

## Discussion

metabolic syndrome is very common in hypertensive population. It has been estimated a prevalence of about 20% in general population and approximately a half in hypertensives, accordingly with our data (4,9,26). This is not surprising, taking into account that the majority of patients with hypertension commonly attended in general practice belong to the cardiovascular high- or very high-risk groups (27). Current European Guidelines establish that an aggressive approach in high-risk hypertensive patients such as those with MetS is mandatory (8). Thus, whether the main purpose in the treatment of hypertension is to achieve an adequate BP control and to reduce the global cardiovascular risk of the hypertensive patient, this is critical in hypertensive patients with MetS (8). Previous studies have shown that BP control is more difficult to achieve, and only about 15% of hypertensive patients with MetS daily attended in Spain are well controlled (9,28). However, our data showed a marked improvement in these figures, with more than 30% of the patients attaining BP goals, especially in lercanidipine group. This is not surprising, as it has been recognised that BP control in Spain has significantly improved in the last years (29).

The efficacy of an antihypertensive drug does not only depend on the capacity to reduce BP values, but on its tolerability profile as well. If a drug is well tolerated, treatment compliance will raise, and secondarily improve BP control (30). While randomised clinical trials are very important to benchmark the effectiveness and tolerability of therapeutic interventions in a controlled scientific manner, they do not always accurately represent the ‘real world’ of clinical practice (31,32). This study was designed in conditions of common clinical practice.

Dihydropyridines have shown to be effective antihypertensive drugs in several clinical trials, but its use has been sometimes limited because of their side effects. The most important adverse events being those related to vasodilatation, especially ankle oedema. However, not all the DHPs have the same tolerability profile. Lercanidipine is a highly lipohilic third generation DHP (11). Its antihypertensive effect results from peripheral vasodilatation and decreased total peripheral resistance (33). This drug has a slow onset of action that helps to avoid reflex tachycardia associated with other DHP. Our data show that incidence and severity of these side effects is significantly higher in the amlodipine/nifedipine group compared with lercanidipine. As BP reductions and concomitant antihypertensive therapy were similar in both groups, this difference in the proportion of adverse events between groups should be explained for a better tolerability profile of lercanidipine. For every group, the increase of dose was associated with a higher incidence of signs and symptoms related to vasodilation suggesting that these side effects are dose dependent. Vasodilatory oedema related to DHP is probably because of an increase in intracapillary hydrostatic pressure that causes fluid filtration from the vascular space to the interstitium. It has been related to an arteriolar dilation that, as a consequence of reflex sympathetic activation, is not accompanied by adequate postcapillary vasodilation (34,35). On the other hand, lercanidipine has shown different effects on plasma norepinephrine levels and a lower sympathetic activation compared with other DHP (36,37), that could, at least in part, explain the lower rate of ankle oedema observed with this drug when compared with amlodipine and nifedipine.

However, the incidence of ankle oedema was high in both groups (42.9% in lercanidipine vs. 55.8% in amlodipine/nifedipine group) and superior to other published data in general hypertensive population (14,20). This is not surprising, taking into account that patients with MetS are polymedicated that significantly increases the risk of presenting side effects (8). Moreover, it is noticeable that the presence of ankle oedema was elicited by using the symptom and signs check list. Thus, it is most likely that a simple heaviness could be interpreted by the patient as ankle oedema that could explain the high incidence, although mild, of that side effect in both groups. On the other hand, when other studies have used the same technique to assess the presence of side effects, the proportion of ARs was very similar (20). In fact, equivalent risk reductions of ankle oedema have been found in other studies when compared lercanidipine with other DHPs (20,38).

In the multivariant analysis, predictors of higher side effects rates were the use of diuretics, that it is not unexpected, as oedemas are commonly treated with diuretics, and the history of cardiac disease, probably because of the intrinsic higher risk of some heart diseases for the development of ankle oedema. Treatment with lercanidipine was a protective factor compared with the use of amlodipine or nifedipine GITS.

This is an observational study with the characteristic design and result limitations of these studies. This methodology reduces the level of control that can be exercised to reduce variation and bias (e.g. random sampling). However, the number of patients included and the nature of the end-points being measured minimises this theoretical limitation. The information derived from this kind of studies aimed to reflect the ‘real world’ of clinical practice appears to be very useful and complementary to the one obtained from the randomised controlled trials. Observational studies include more often older patients with a higher comorbidity that, in terms of drug tolerability, could reflect the ‘real world’ clinical scenario better than randomised controlled trials. The method used to evaluate compliance is the self-communicated interview as indicated by Haynes–Sackett test. Despite its known limitations, it has been shown that this test can determine adequately the treatment compliance in clinical practice (25,39).

In conclusion, lercanidipine appears to exhibit a better tolerability profile and less vasodilation-related ARs compared with other DHPs in hypertensive patients with MetS.
